# Interferon-γ signal drives differentiation of T-bet^hi^ atypical memory B cells into plasma cells following *Plasmodium vivax* infection

**DOI:** 10.1038/s41598-022-08976-6

**Published:** 2022-03-22

**Authors:** Piyawan Kochayoo, Pongsakorn Thawornpan, Kittikorn Wangriatisak, Siriruk Changrob, Chaniya Leepiyasakulchai, Ladawan Khowawisetsut, John H. Adams, Patchanee Chootong

**Affiliations:** 1 Department of Clinical Microbiology and Applied Technology, Faculty of Medical Technology, Mahidol University, Bangkok, 10700 Thailand; 2 Department of Parasitology, Faculty of Medicine Siriraj Hospital, Mahidol University, Bangkok, 10700 Thailand; 3 Department of Global Health, University of South Florida, Tampa, FL 33612 USA

**Keywords:** Malaria, Parasite host response

## Abstract

For development of a long-lasting protective malaria vaccine, it is crucial to understand whether *Plasmodium*-induced memory B cells (MBCs) or plasma cells develop and stably contribute to protective immunity, or on the contrary the parasite suppresses antibody responses by inducing MBC dysfunction. The expansion of T-bet^hi^ atypical MBCs is described in chronic *Plasmodium falciparum*-exposed individuals. However, it remains unclear whether accumulation of T-bet^hi^ atypical MBCs is indicative of a protective role or rather an impaired function of the immune system in malaria. Here, the phenotypic and functional features of T-bet^hi^ atypical MBCs were studied in *P. vivax* patients living in an area of low malaria transmission. During *P. vivax* infection, the patients produced a twofold higher frequency of T-bet^hi^ atypical MBCs compared to malaria non-exposed individuals. This distinct atypical MBC subset had a switched IgG phenotype with overexpression of activation markers and FcRL5, and decreased Syk phosphorylation upon BCR stimulation. Post-infection, expansion of T-bet^hi^ IgG^+^ atypical MBCs was maintained for at least 3 months. Further studies of the contribution of T-bet^hi^ atypical MBC function to humoral immunity showed that synergizing IFN-γ with TLR7/8 and IL-21 signals was required for their differentiation into plasma cells and antibody secretion.

## Introduction

Antibodies play an essential role in protection against malaria. Anti-*Plasmodium* antibodies prevent disease progression in several ways: (i) block sporozoites from invading hepatocytes^1, 2^; (ii) opsonize merozoites and activate cell-mediated death; (iii) prevent erythrocyte invasion and block Plasmodial proteins from binding to molecules on cell surfaces^2^. However, the anti-*Plasmodium* antibodies acquired following infection decline substantially within a few years in the absence of re-infection^3, 4^, and boost inconsistently upon antigen re-exposure^5, 6^. These impairments of long-lived MBC or plasma cell responses may contribute to an inefficient protective immunity against malaria.

Malaria infection triggers the response of *Plasmodium*-specific MBCs as reported in several field studies^7–11^. A controversy remains about the development and persistence of MBCs, whether their responses are short-or long-lived. Individuals residing in areas of high transmission appear to develop a low frequency of malaria-specific MBCs, even when parasite loads are sufficient and capable of inducing antibody responses after infection^8, 10^. However, long-lived responses of *Plasmodium*-specific MBCs are reported in areas of low malaria endemicity, and stable *Plasmodium*-specific MBCs occur without frequent boosting^9, 11^. These contrasting reports of MBC responses to malaria are probably influenced by differences in intensity of malaria transmission and nature of the antigens expressed, as well as by age and host genetics. More immuno-epidemiological studies of MBC responses in malaria endemic and epidemic populations may better inform the design of malaria vaccines.

Transcription factor T-bet plays critical roles as a transcriptional regulator in several immune lineages, including interferon γ (IFN-γ)-secreting CD4^+^ Th1 cells and B cells^12, 13^. T-bet^+^ B cells are found in both mice and humans^12^. They promote antibody class switching to IgG2a/c in murine^14, 15^, and IgG1/IgG3 in human B cells^16, 17^, and control viral infections^18, 19^. T-bet^+^ B cells that are CD21^−^CD27^−^ are phenotypically similar to atypical MBCs. In chronic immune activation, this cell population diminishes B cell receptor (BCR) signaling and limits antibody production^17, 20, 21^. Although T-bet^hi^ atypical MBCs are shown to be exhausted cells in chronic antigenic stimulation, they appear to play a role in autoantibody secretion^22^, functional responses to yellow fever, vaccinia and influenza virus vaccination, as well as primary HIV infection^16, 23^. *P. falciparum*-exposed individuals from moderate to high malaria transmission areas produce a high number of T-bet^hi^ atypical MBCs^17, 24, 25^. The booster response of this cell population was shown in a human immunization study^26^. At present, atypical MBCs are proposed to potentially function as: (i) pre-antibody secreting cells (pre-ASCs)^22, 27^, (ii) activated B cells in normal immune responses to vaccination and some infections^16, 26^, (iii) immune regulators which upregulate FcRL5 inhibitory receptor in the presence of high IgG levels^24^, or (iv) anergic MBCs that contribute to inefficient humoral immunity^17, 21^. Despite extensive data showing an expansion of atypical MBCs with malaria exposure, the exact role of these cells under transcription T-bet control remains incompletely understood. Most of reports are documented in *P. falciparum* malaria but lacking in *P. vivax*, which has unique biological characteristics such as dormant liver-stages and relapsing fever. Given interspecies variation, their functional roles in triggering of immune responses might be different. Future studies of the phenotypic characteristics, dynamic responses, and functions of atypical MBCs in geographical settings with varying malaria transmission intensities, as well as the drivers of their stimulation and differentiation, are required to clarify their role in humoral immunity.

Here, the frequency, phenotypic characteristics and kinetics of T-bet^hi^ atypical MBCs were analysed in *P. vivax*-infected patients in a low transmission intensity setting. The function of T-bet^hi^ B cells (atypical MBCs), and the signals that induce their differentiation into plasma cells, were evaluated in these patients. Our findings may benefit the further development of efficacious malaria vaccines.

## Results

### Increased proportion of T-bet^hi^ atypical MBCs during acute ***P. vivax*** infection

**Figure 1:**
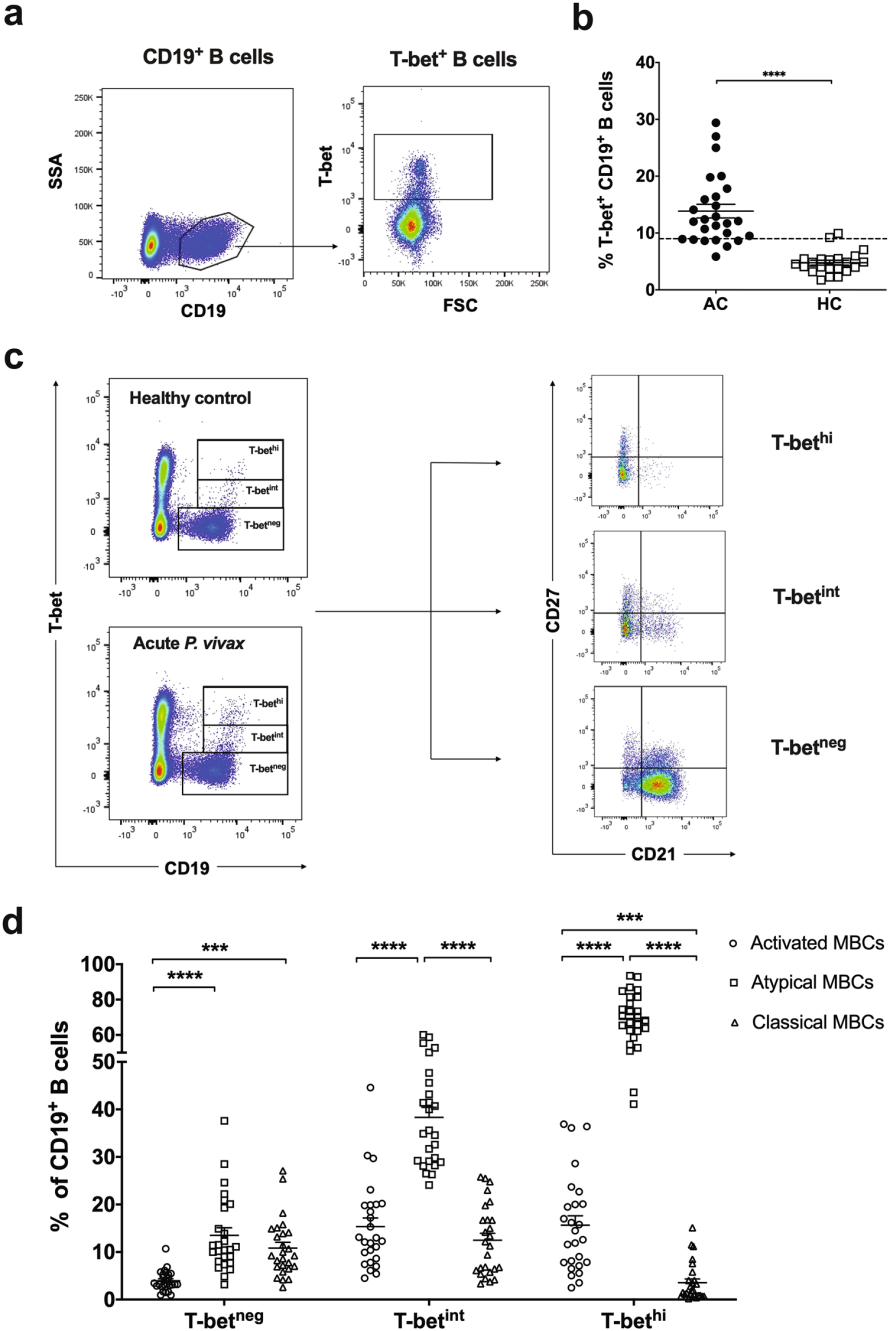
Increased proportion of T-bet^hi^ atypical MBCs during acute *P. vivax* infection. (**a**) Representative flow cytometry plot indicating expression of T-bet in CD19^+^ B cells. (**b**) The frequency of T-bet^+^ B cells is shown from acute *P. vivax* patients (AC; *n* = 26) compared to healthy controls (HCs; *n* = 22). (**c**) Representative flow cytometry plot indicating profiling expression of T-bet, basing on low, intermediate or high expression in total CD19^+^ B cells. Among T-bet^neg^, T-bet^int^ and T-bet^hi^ B cells, activated, classical and atypical MBCs were defined. (**d**) The frequency of each subpopulation was determined. The horizontal lines represent mean values ± SEM. Statistical analyses were performed using Mann–Whitney *U* tests (for unpaired variables) and Wilcoxon signed rank tests (for paired variables). ***P < 0.001; ****P < 0.0001.

To observe the acquisition of T-bet^hi^ atypical MBC responses in natural *P. vivax* infection in the setting of low-level transmission, the frequency of T-bet^+^ B cells was determined during acute malaria. Most (85%) subjects possessed a significantly higher frequency of these cells than did healthy controls (HCs) (Fig. 1a, b and Supplementary Table S1). As the T-bet^**+**^ MBC population is heterogeneous^17, 28, 29^, to understand their function as contributors to antibody responses, T-bet profiling (based on low, intermediate, and high expression on CD19^+^ B cells and among MBC subsets) was determined (Fig. 1c). Of T-bet^hi^ B cells, 69.31%, 15.64% and 3.55% exhibited atypical, activated and classical MBC phenotypes, respectively. T-bet^int^ B cells made up 38.33% of atypical, 15.36% of activated, and 12.50% of classical MBCs. In addition, among T-bet^neg^ B cell subsets, 13.52% were atypical, 3.95% were activated, and 10.85% were classical MBCs (Fig. 1d).

### T-bet^hi^ atypical MBCs were skewed toward IgG class switching

**Figure 2:**
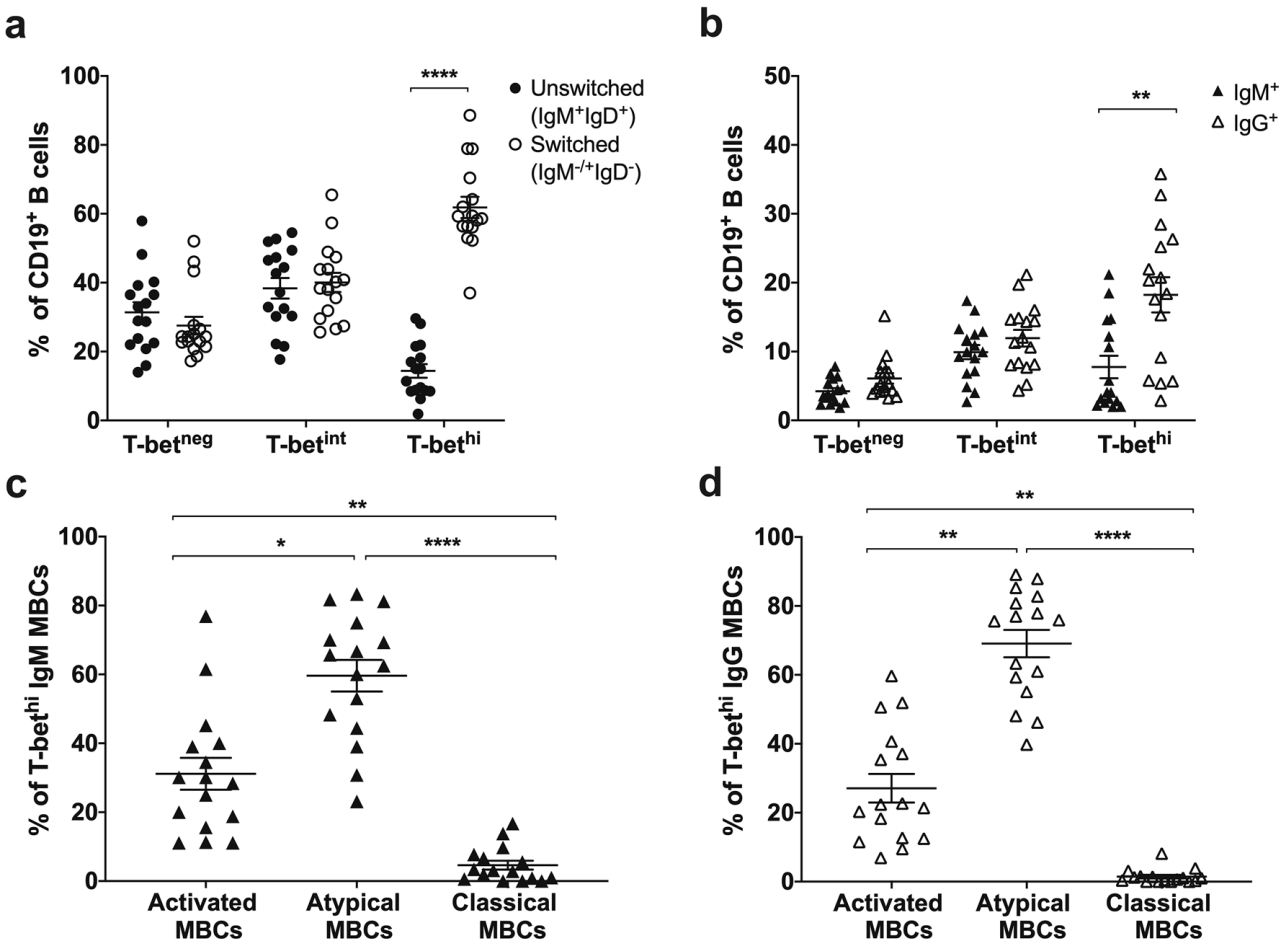
T-bet^hi^ atypical MBCs were skewed toward IgG class switching. (**a**) The frequencies of unswitched (IgM^+^IgD^+^) and switched (IgM^+/−^IgD^−^) MBCs were detected during acute disease (*n* = 16). (**b**) The frequencies of T-bet^hi^ IgM and IgG MBCs was also detected in these patients. Three subsets of MBCs (activated, atypical and classical) were detected among (**c**) T-bet^hi^ IgM and (**d**) IgG MBCs. The horizontal lines represent mean values ± SEM. Statistical analyses were performed using Wilcoxon signed rank tests; *P < 0.05; **P < 0.01; ****P < 0.0001.

We investigated whether the expanded T-bet^hi^ atypical MBCs during acute *P. vivax* infection mainly expressed switched or unswitched MBC phenotypes. Of the 26 samples from acutely infected patients, 16 were initially detected of IgM and IgD expression in the T-bet^hi^ B cells (Supplementary Fig. 1 and Supplementary Table S1). There was a higher percentage (62%) of T-bet^hi^ switched (IgM^+/−^IgD^−^) than unswitched (IgM^+^, IgD^+^) MBCs (Fig. 2a). We next addressed whether the T-bet^hi^ switched MBCs were to IgM or IgG. Significantly more IgG than IgM was detected in this population (Fig. 2b). Further analysis of T-bet^hi^ switched (IgM or IgG) MBCs (from CD21, CD27 expression) showed that the phenotypic markers were mainly on atypical MBCs (T-bet^hi^ IgM; average 59.63%, SEM 4.58%; T-bet^hi^ IgG; average 69.07%, SEM 3.97%) and activated MBCs (T-bet^hi^ IgM; average 31.16%, SEM 4.62%; T-bet^hi^ IgG 27.10%, SEM 4.17%) (Fig. 2c, d).

### Expansion of T-bet^hi^ atypical MBCs was stably maintained post-infection

**Figure 3:**
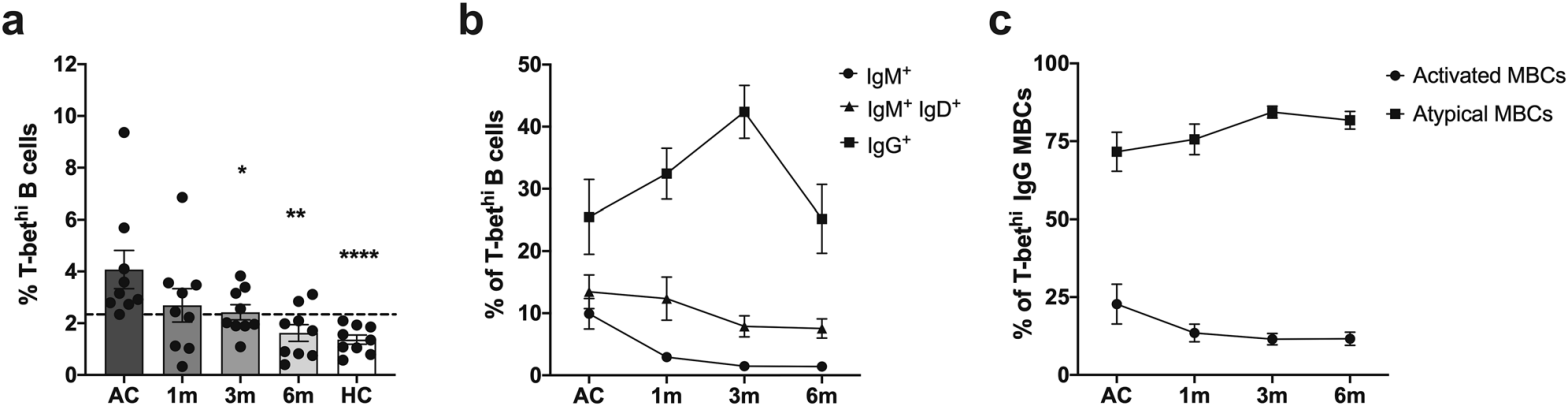
The expansion of T-bet^hi^ atypical MBCs was stably maintained after infection. (**a**) The frequency of T-bet^hi^ B cells from 9 acutely *P. vivax*-infected patients compared at three follow-up times (1, 3 and 6 months after infection), along with those of healthy controls (HCs; *n* = 9). The horizontal lines represent mean values ± SEM. Dashed line indicates the base line of HCs (average + 2SD). (**b**) The frequency T-bet^hi^ unswitched (IgM^+^IgD^+^) and switched (IgM and IgG) MBCs. (**c**) The frequency of T-bet^hi^ IgG activated and atypical MBCs at each time point is shown as mean values ± SEM of all subjects. The horizontal lines represent mean values ± SEM. Statistical analyses were performed using Mann–Whitney *U* tests; *P < 0.05; **P < 0.01; ****P < 0.0001.

We observed kinetic responses of T-bet^hi^ B cells and their subsets during and after *P. vivax* infection. Of the 26 patients, 9 with higher frequencies of T-bet^hi^ B cells than did HCs (2.34%; average + 2SD of HCs) were recruited as a cohort for tracking changes in the cell frequencies during acute malaria (AC) and after infection (Supplementary Table S1). During AC, all patients in this cohort presented higher total T-bet^hi^ B cells (average, 4.42%; SEM 0.73%) compared to the percentage in HCs (average 1.27%; SEM 0.18%). Post-infection, the population of T-bet^hi^ B cells expanded further as found at the 1-month sampling (average 2.59%; SEM 0.65%). However, the frequency of these cells was significantly decreased at the 3-month post-infection time-point compared to AC (Fig. 3a). Four and 2 patients maintained elevated T-bet^hi^ levels 3 and 6 months after infection, respectively (Fig. 3a).

The tracking of unswitched (IgM^+^IgD^+^), switched IgM and switched IgG MBCs among T-bet^hi^ B cells showed that the switched IgG MBCs found during AC (average 25.51%; SEM 6.02%) were further increased 3-month post-infection (average 42.39%; SEM 4.26%) and then declined to baseline levels 6 months after parasite clearance (average 25.19%; SEM 5.53%). In contrast, much lower frequencies of T-bet^hi^ switched IgM (average 9.92%; SEM 2.47%) and unswitched MBCs (average 13.46%; SEM 2.72%) were observed during AC. These tended to decrease at 1-month post-infection (T-bet^hi^ switched IgM; average 2.93%; SEM 0.77% and unswitched MBCs; average 12.34%; SEM 3.49%) and continued to decline over subsequent timepoints, indicating that T-bet^hi^ unswitched MBCs were likely switched into T-bet^hi^ IgG MBCs after infection (Fig. 3b and Supplementary Fig. 2). Further kinetic analysis of T-bet^hi^ IgG atypical MBCs showed that cell frequency during AC (average 71.61%; SEM 6.26%) remained stably detectable at all follow-up times with no significant difference of those frequencies through the recovery phase. However, the frequency of T-bet^hi^ IgG activated MBCs (average 22.76%; SEM 6.41%) was relatively low and stayed so to the 6-month blood collection (Fig. 3c and Supplementary Fig. 3).

### T-bet^hi^ atypical MBCs from acute ***P. vivax*** patients had upregulated activation markers and FcRL5

**Figure 4:**
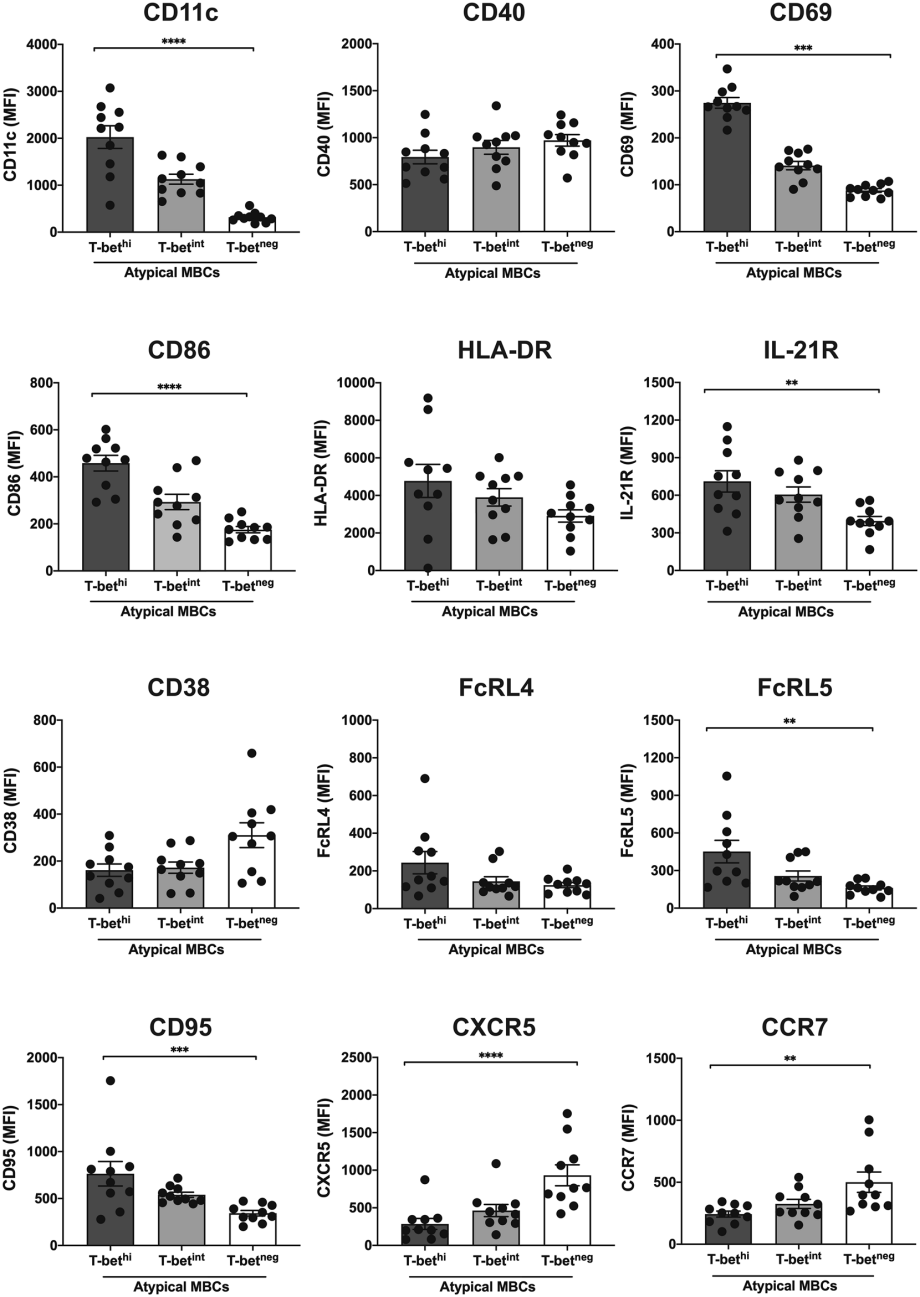
T-bet^hi^ atypical MBCs from acute *P. vivax* patients had upregulated activation markers and FcRL5. Ex vivo expression of cell surface protein on CD19^+^ B cells stratified by level of T-bet expression in acute patients (*n* = 10). The horizontal lines represent mean values ± SEM. Statistical analyses were performed using Wilcoxon signed rank tests; **P < 0.01; ***P < 0.001; ****P < 0.0001.

To understand whether and how T-bet^hi^ atypical MBCs participate in immune responses to acute *P. vivax* infection, we further characterized the ex vivo functional profile of these MBCs by flow cytometry for inhibition markers (FcRL4, FcRL5 and CD95), activation markers (CD11c, CD40, CD69, CD86, HLA-DR and IL-21R) and chemokine receptor (CXCR5 and CCR7) expression in independent acute vivax malaria patients (*n* = 10) (Fig. 4). Significantly higher expression of CD11c, CD69, CD86, IL-21R, FcRL5 and CD95 was detected in T-bet^hi^ atypical MBCs compared to T-bet^neg^ atypical MBCs, whereas significant decreases of expression of chemokine receptors CXCR5 and CCR7 were observed in T-bet^hi^ atypical MBCs (Fig. 4).

### T-bet^hi^ atypical MBCs of ***P. vivax***-infected patients greatly reduced Syk BCR signaling

**Figure 5:**
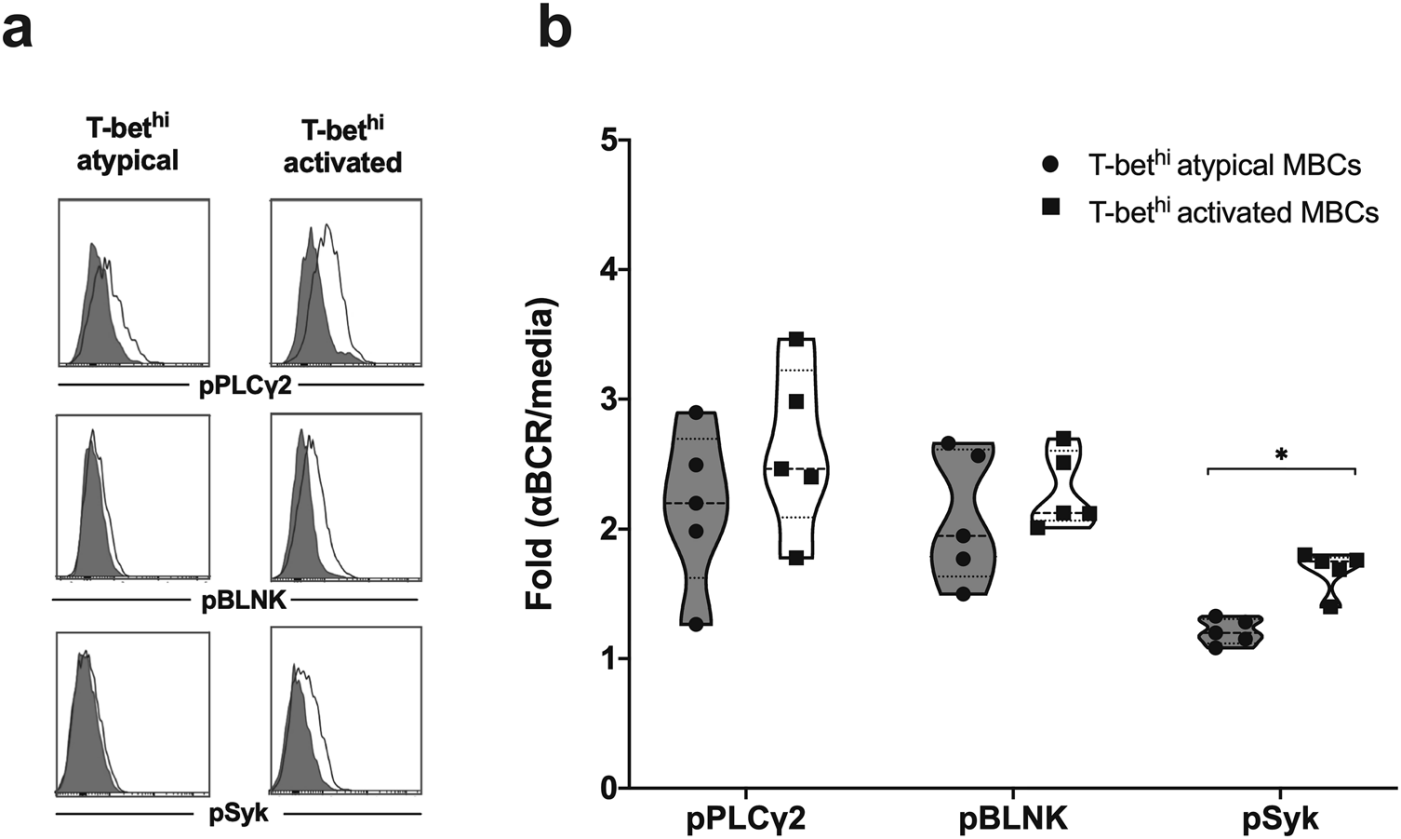
T-bet^hi^ atypical MBCs of *P. vivax*-infected patients reduced BCR signaling. (**a**) Representative histograms indicating expression of pSyk, pBLNK, and pPLCγ2 in MBC subsets cross-linked for 5 min with anti-IgM/IgG Abs (black line tracing) and uncross-linked (shaded gray area). (**b**) Mean fluorescence intensity (MFI) of pSyk, pBLNK, and pPLCγ2 in T-bet^hi^ activated and atypical MBCs after BCR cross-linking (*n* = 5). The horizontal lines represent mean values ± SEM. Statistical analyses were performed using Wilcoxon signed rank tests; *P < 0.05.

As noted above, T-bet^hi^ atypical MBCs upregulated both activation and inhibition markers. To observe whether the upregulated markers on these MBCs are related to upstream BCR signaling events, the phosphorylation of BCR signaling molecules was investigated in patients. We found a significant decrease of Syk phosphorylation in T-bet^hi^ atypical compared to T-bet^hi^ activated MBCs following BCR cross-linking, while slight reductions of pPLCγ2 and pBLNK BCR molecules were observed in these MBCs (Fig. 5a, b). In addition, BCR-induced pSyk in T-bet^hi^ atypical MBCs of *P. vivax* patients was lower than in HCs, whereas no difference was observed in T-bet^hi^ activated nor classical MBCs (data not shown). Further investigation of functional capacity of these T-bet^hi^ atypical MBCs to produce antibody is needed to determine whether similar or different signals from classical MBCs are required for their effector function.

### IFN-γ synergizes with TLR7/8 and IL-21 to drive T-bet^hi^ atypical MBC differentiation into plasma cells

**Figure 6:**
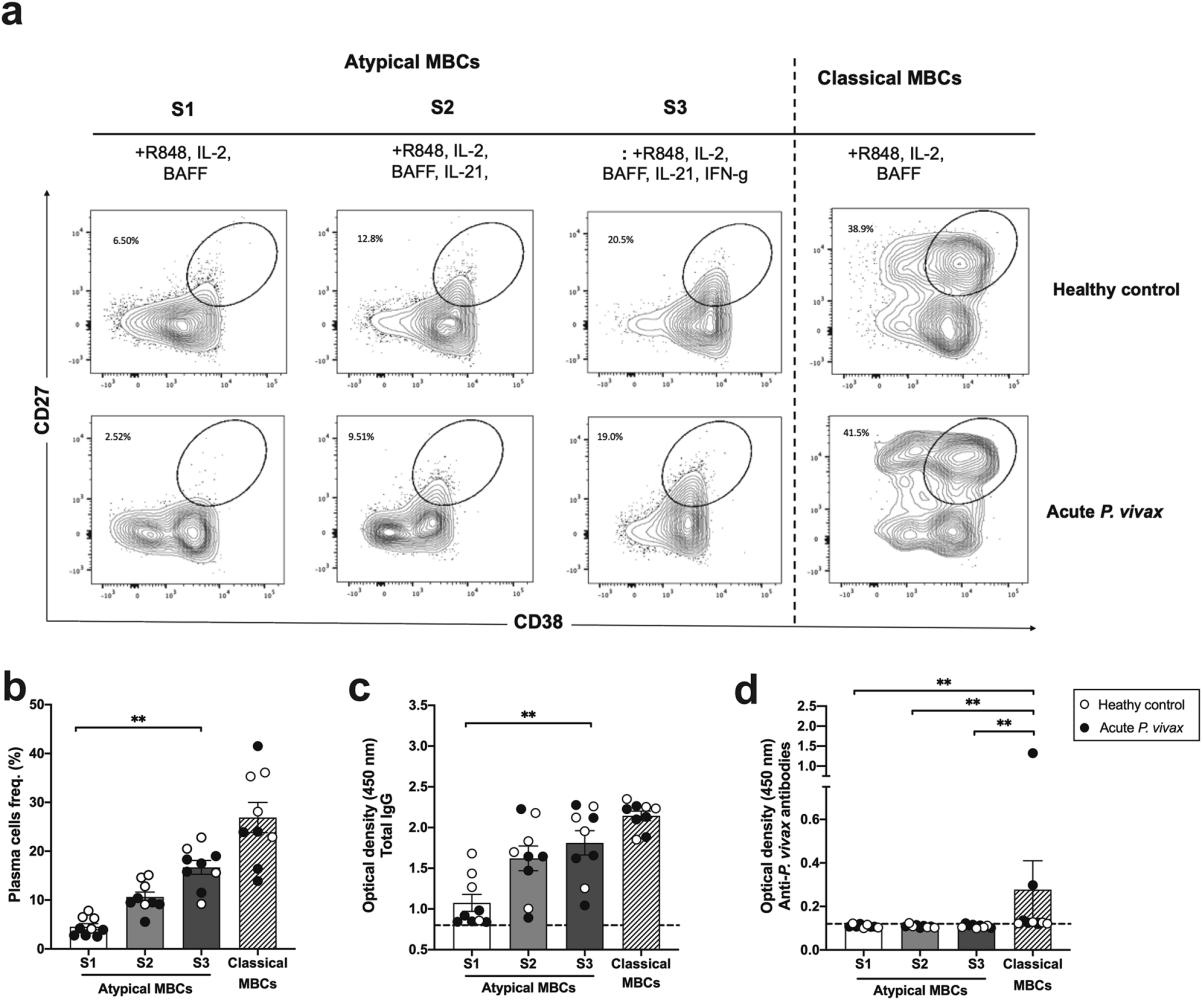
IFN-γ synergizes with TLR7/8 and IL-21 to induce T-bet^hi^ atypical MBC differentiation into plasma cells. (**a**) Representative flow cytometry plot indicating expression of plasma cell differentiation markers (CD27^+^, CD38^+^) from atypical MBC cultures with 3 conditions of stimuli (Stimuli 1 (S1): R848, IL-2 and BAFF; S2: R848, IL-2, BAFF and IL-21; S3: R848, IL-2, BAFF, IL-21 and IFN-γ), as well as classical MBC cultures, are shown for healthy controls and acute *P. vivax* patients. (**b**) Frequencies of plasma cells, (**c**) optical density of total IgG and (**d**) anti-*P. vivax* specific antibodies were determined in each culture condition. The horizontal lines represent mean values ± SEM. Statistical analyses were performed using Mann–Whitney *U* tests (for unpaired variables) and Wilcoxon signed rank tests (for paired variables); **P < 0.01. White circles refer to healthy controls and black circles refer to acute *P. vivax* patients.

As T-bet^hi^ atypical MBCs showed reductions of upstream BCR signaling events, their differentiation into plasma cells and their secretion of antibody were further assessed in the acute *P. vivax* patients. We initially stimulated T-bet^hi^ atypical MBCs (atypical MBCs) under conditions which readily drove classical MBCs into plasma cells, stimulation in vitro with TLR7/8 (R848) and B-cell activating factors (IL-2 and BAFF)^21^. Our results showed that classical MBCs could differentiate into plasma cells in both patients (average 23.94%; SEM 4.83%) and HCs (average 30.60%; SEM 3.13%). For atypical MBCs, 6.17% of plasma cells were detected in HC cultures, whereas fewer (3.22%) were found in *P. vivax* patients, indicating that atypical MBCs might require more signals to generate plasma cells (Fig. 6a, b).

Our data showed that T-bet^hi^ atypical MBCs displayed upregulated IL-21R during acute *P. vivax* malaria (Fig. 4). We next observed an ability of IL-21, which are T follicular helper (Tfh)-derived signals, to promote the differentiation of these cells in vitro. The combination of IL-21 with TLR7/8, IL-2 and BAFF resulted in approximately 2.5-fold higher induction of atypical MBCs into plasma cells (average 8.82%; SEM 0.84%) compared to the same condition without IL-21. Compared with HCs, atypical MBCs from patients had a slightly lower ability to generate plasma cells (HC; average 12.90%; SEM 1.36%) (Fig. 6a, b), suggesting that the IL-21 signal could help in their development. Since IFN-γ signal was reported to drive double negative 2 (DN2) (CD27^−^, IgD^−^, CXCR5^−^ and CD11c^+^) B cell differentiation into plasma cells in autoimmune diseases following stimulation with TLR7/8 and IL-21^22, 27^, we next included IFN-γ, Th1-derived signals, in atypical MBC culture to enhance their differentiation. In the presence of IFN-γ, atypical MBCs from *P. vivax* patients and HCs generated twofold more plasma cells compared to the same condition without IFN-γ stimulation (Fig. 6a, b), indicating that atypical MBCs required signal from Th1 cells to promote differentiation into plasma cells.

In addition, the functional ability of atypical MBCs to secrete antibody was observed upon stimulation with various signals. Upon TLR7/8, IL-2 and BAFF stimulation, total IgG was greatly detected in classical MBC culture supernatant, whereas these signals did not induce antibody secretion from atypical MBCs (Fig. 6c). Addition of T-cell-derived cytokines into atypical MBC cultures (TLR7/8, IL-2 and BAFF) showed that IL-21 increased total IgG antibodies and IFN-γ significantly increased IgG levels in supernatant (Fig. 6c). Importantly, *P. vivax* antigen-specific antibody was specifically detected in classical MBC cultures, from 2 of 5 patients. The undetectable level of anti-*P. vivax* antibody upon TLR7/8, IL-21 and IFN-γ stimulation of atypical MBCs was observed due to the low frequency of antigen specific atypical MBCs in blood circulation (Fig. 6d).

## Discussion

Expansion of T-bet^+^ B cells has been described in the context of various chronic antigenic stimulations, including aging^30, 31^, autoimmunity^27, 32^, vaccination^16, 23^, and infectious diseases such as HIV, HCV, and *P. falciparum* malaria^16, 17, 29^. This cell population expressed the phenotype of as atypical MBCs (CD21^−^CD27^−^), and also expressed CD11c, CXCR3 and FcRL5^17, 25, 26^. However, the function of high expression of T-bet in atypical MBCs in these models, as well as the triggers of their differentiation into plasma cells, is unclear. Our results revealed an increase of T-bet^hi^ B cells during acute *P. vivax* infection. Most of the responses were T-bet^hi^ atypical MBCs with IgG class switching, and were stably maintained the responses in the convalescence phase for at least 3 months. These T-bet^hi^ atypical MBCs overexpressed CD11c, CD69, CD86 and IL-21R, as well as FcRL5 and CD95. Functional analysis of these MBCs exhibited diminished BCR signaling following cross-linking. To drive the functional role of T-bet^hi^ atypical MBC differentiation into plasma cells and antibody secretion, stimulation with a combination of TLR-7/8 and T cell-derived cytokines (IL-21 and IFN-γ) was required. Together, our data suggest that *P. vivax* infection triggered an expansion of T-bet^hi^ atypical MBCs and that these expanded cells played a role in anti-malarial humoral immunity under TLR7/8 ligands, and Tfh- and Th1-derived signals.

A more detailed understanding of the generation and persistence of the atypical MBCs associated with malaria infection is useful for vaccine development. In *P. falciparum* malaria, an increase of T-bet^hi^ atypical MBCs occurs in children and adults living in moderate and high transmission settings. These expanded cells were commonly found to express CD11c^+^, CXCR3^+^, FcRL5^+^ phenotypes and exhibited reduced BCR signaling^17, 21, 24^. Moreover, the number of atypical MBCs is higher in children with chronic asymptomatic *P. falciparum* infection^17, 21^ or previous exposure to the parasite compared to having a primary infection^25^. These previous findings suggest that exposure to parasite drives expansion of T-bet^hi^ atypical MBCs and that the chronic presence of parasites induces accumulation of this MBC subset with reduced B cell effector function. Here, we observed the functional characteristics of T-bet^hi^ atypical MBCs in *P. vivax* subjects who lived in an area of Thailand with seasonal malaria transmission (a single rainy season peak). During acute malaria, T-bet^hi^ atypical MBCs were expanded, mainly switched to IgG subclass, and had elevated expression of activation markers (CD11c, CD69, CD86, IL-21R), FcRL5 and CD95. Moreover, CXCR5 and CCR7 were downregulated among the T-bet^hi^ atypical MBCs. After parasite clearance, the switched IgG^+^ T-bet^hi^ atypical MBCs persisted for at least 3 months. Our data proved that non-chronic infection to *P. vivax* infection drives an expansion of T-bet^hi^ atypical MBCs which exhibit T-dependent responses such as upregulation of activation markers and IL-21R. A possible explanation of the T-bet^hi^ atypical MBC response with IL-21R^+^ phenotype in vivax malaria is that B cells encounter parasite antigen via BCRs and receive signals from CD4^+^ T cells which subsequently drive migration of B cells into germinal centers (GCs)^33, 34^. Within these GCs, T-B interactions may induce upregulation of IL-21R on B cells, which in turn supports the activation of IL-21 signaling in GCs. Tfh cells, with polarizing IFN-γ and IL-21, contribute to the upregulation of T-bet and development of an atypical phenotype (CD21^−^CD27^−^) on these cells. In addition, they potentially participate in isotype switching to IgG and persist after parasite clearance^17, 33, 35^. The lower expression of the GC-positioning receptors CXCR5 and CCR7 on these cells might explain their migration outside GCs to play a crucial role in humoral immunity. The overexpression of FcRL5 on IgG^+^ T-bet^hi^ atypical MBCs in vivax malaria might be part of a feedback mechanism which downregulates the hyperactivated state of atypical MBCs, or they may be markers of durable and robust responses to re-infection as previously proposed in *P. falciparum* studies^36^. In-depth investigations regarding these pivotal signals of the immune mechanisms that drive T-bet^hi^ atypical MBCs to play effector function are still needed for improved malaria vaccine design.

The role of T-bet^hi^ atypical MBCs in the immune system remains elusive. To date, the signals inducing high expression of T-bet in atypical MBCs, as well as the function of this cell population in the immunity, require more understanding. In malaria, it has been reported that *P. falciparum-*infected erythrocytes drive expansion of T-bet in B cells^37^, and that IFN-γ is the key driver for T-bet^hi^ atypical MBCs differentiation^17, 20^. With these findings, a functional role of T-bet^hi^ atypical MBCs might be immunosuppression, as suggested by the upregulation of multiple inhibitory receptors with reduced BCR-mediated signaling and limited antibody production^17, 20, 21^. Up to now, there is no report in *P. vivax* malaria which differs from *P. falciparum* since it has a dormant stage in the human liver (hypnozoites) and a relapsing fever. Moreover, different malaria transmission settings may influence the functional roles of atypical MBCs. Here, we found that T-bet^hi^ atypical MBCs from *P. vivax*-infected patients had significantly reduced Syk BCR signaling upon cross-linking. This indicates that these cells might have less activation in response to BCR signaling or require additional signals for transition from resting to a highly active state. Our functional analysis focused on antibody responses demonstrated that, unlike classical MBCs, atypical MBCs did not differentiate into plasma cells and secrete IgG antibodies upon TLR7/8, IL-2 and BAFF stimulation. These results suggested that atypical MBCs require different or more signals for plasma cell generation. Previous reports documented that T cell-derived cytokines (IL-21 and IFN-γ) were significantly increased in sera from patients with autoimmune diseases^38, 39^ and *P. falciparum* malaria infection^40, 41^. Of note, we found upregulation of IL-21R on these cells. The addition of IL-21 to atypical MBCs increased the capacity to induce plasma cell generation, indicating that IL-21 is one of the most potent cytokines derived from Tfh cells in regulating atypical MBCs. Furthermore, we observed their functional capacity by adding Th1-derived cytokines. Our results showed that plasma cell differentiation was enhanced by IFN-γ, as it showed a higher frequency of plasma cells in atypical MBC culture compared to the condition without IFN-γ. Collectively, the findings indicated that atypical MBCs required multiple stimuli including TLR7/8, IL-21 and IFN-γ signals, to induce plasma cell generation. Of note, anti-*P. vivax* specific antibodies were found in classical MBC cultures of some patients, but not in atypical MBC cultures, likely reflecting the low level of *P. vivax* specific atypical MBCs in circulating blood. Thus, there is a need to develop high sensitivity techniques for detection of the rare population of malarial specific MBCs in producing anti-malarial specific antibodies as well as their protective roles in further studies.

For the studies of MBC responses in malaria, the complex life cycle, antigenic variation/polymorphism, and its ability to establish chronic infection could well influence development and longevity of MBC responses. *P. vivax* parasites have unique biological features compared to *P. falciparum*. The main differences of *P.* vivax parasite include: (i) dormant hypnozoite in liver, which often escapes immune response and leads to latent state^42, 43^, (ii) recurrent parasitemia implicating in relapsing symptoms^44^, (iii) preference for invading reticulocytes and increasing their deformability and fragility leading to evasion of immune system^45, 46^. Given these different characteristics, the detailed knowledge of atypical MBC function in *P. falciparum* infection might not explain in *P. vivax*-exposed individuals. In comparison of T-bet^hi^ atypical MBC responses in malaria, these cells were increased in frequency and activated by showing upregulation of activating markers (CD11c, CD69) and co-stimulatory molecules (CD86 and IL-21R) in both *P. falciparum and P. vivax* infection^17, 20, 47^. For the contribution of atypical MBCs in humoral immunity, the BCR signaling (PLCγ2, pBLNK and Syk) of *P. falciparum*-associated atypical MBCs was impaired^21^, thereby requiring *Staphylococcal enterotoxin B* and Tfh cell interaction for plasma cell differentiation^47^. In *P. vivax*, only greatly reduced Syk BCR signaling was detected in T-bet^hi^ atypical MBCs from infected patients, resulting in requirement of additional signals from TLR7/8 and T cell-derived cytokines (IL-21 and IFN-γ) for driving into plasma cell differentiation and antibody secretion. Future exploration is required to determine the role of Tfh cells in contribution to activation and differentiation of T-bet^hi^ atypical MBCs into plasma cells.

Limitation of this study is a small sample size for tracking kinetic responses of expanded T-bet^hi^ atypical MBCs in cohort analysis. Nine of total 26 who had higher T-bet^hi^ atypical MBC frequencies than HCs (average + 2 SDs of HCs) were recruited in a 6-month cohort study. Thus, this observation should be considered as preliminary result to demonstrate the maintenance of T-bet^hi^ IgG atypical MBCs in response to *P. vivax* infection. More screening of expanded T-bet^hi^ atypical MBCs samples at acute disease and cohort study design would help to ensure their kinetic responses after parasite clearance as well as to clarify their functional roles in malaria immunity.

In summary, *P. vivax* infection was found to be associated with T-bet^+^ B cell activation and expansion. The predominant response was of T-bet^hi^ atypical MBCs with an IgG phenotype. These cells showed a profile of upregulated activation markers and FcRL5, and exhibited reduced responsiveness to pSyk following cross-linking. Notably, the signals from TLR7/8 and T cell-derived cytokines (IL-21 and IFN-γ) appeared to drive their function in anti-malarial humoral immunity. A better understanding of the regulators and functions of these expanded cells, in settings of chronic stimulation by various antigens, will provide further insight into the mechanism of their differentiation, and may improve vaccine strategies and therapeutic interventions.

## Methods

### Ethical statement

Ethical approval was obtained from the Committee on Human Rights Related to Human Experimentation, Mahidol University, Thailand (MUIRB 2012/079.2408). The study participants gave written informed consent to enroll in this study before collecting blood samples. All experiments involving human subject were conducted in accordance with relevant guidelines and regulations.

### Study subjects and sampling

Twenty-six patients with acute *P. vivax* malaria living in an area of low intensity malaria transmission (Kraburi, Ranong Province, a village near the Myanmar border in Southern Thailand), were enrolled in the study for determination of frequency and phenotype of T-bet^hi^ atypical MBCs. Of the 26, the 9 who had T-bet^hi^ atypical MBC frequencies more than the average + 2 standard deviations (SDs) of that in HCs were enrolled in a 6-month cohort sub-study to track the kinetic changes of these cells with follow-up visits at 1, 3 and 6 months after infection. To further explore the profile of T-bet^hi^ atypical MBCs during *P. vivax* infection, 10 subjects were recruited for analysis of activation and inhibition marker expression. The function of T-bet^hi^ atypical MBCs was analysed for upregulation of BCR signaling molecules, differentiation into total IgG, and anti-*P. vivax* antigen-specific antibodies after in vitro stimulation (*n* = 5).

*Plasmodium vivax* infection was confirmed by microscopic examination and nested PCR of peripheral blood. Patients were evaluated for sub-patent parasitemia every 2 weeks. Data on past malaria infections were obtained from the records of the Vector-borne Disease Unit. Twenty-six malaria non-infected Thai residents who lived in non-endemic areas (Bangkok, Thailand) were recruited as HCs.

### Flow cytometry

Flow cytometry of peripheral blood mononuclear cells (PBMCs) was performed as previously described^7^. Briefly, PBMCs were incubated with Live/Dead fixable stain (Invitrogen, USA) and monoclonal antibodies (mAbs) (Supplementary Table S2). Cells were then fixed and permeabilized according to the manufacture’s instructions (Transcription Factor Buffer Set, Biolegend, USA). Intracellular targets were stained with a transcription factor T-bet. Analyses were done with a BD FACS Canto II flow cytometer (BD Biosciences, USA).

### BCR signaling assay

Two million PBMCs were stained with anti-CD19, -CD21 and -CD27 mAbs. Cells were washed and incubated at 37 °C for 30 min before adding F(ab’)2 anti-IgM and anti-IgG (Southern Biotech and Jackson ImmunoResearch, USA) at a final concentration of 20 μg/ml and incubated at 37 °C for 5 min. For detection of phosphoproteins, cells were fixed, permeabilized and stained with antibodies specific for phospho-Syk (Y352) (pSyk), pBLNK (Y84) and pPLCγ2 (Y759) (BD Biosciences) and T-bet (Supplementary Table S2).

### B cell sorting and stimulation

B cell subsets were sorted using FACS Aria III (BD Biosciences). The gating strategy of cell sorting was shown in Supplementary Fig. 4. Sorted cells (5 × 10^4^) were cultured in RPMI media supplemented with 3 conditions of stimuli; Stimuli 1 (S1): 1 μg/ml R848 (Invivogen, USA), 10 ng/ml IL-2 (Peprotech, USA), 10 ng/ml BAFF (Peprotech); S2: 1 μg/ml R848, 10 ng/ml IL-2, 10 ng/ml BAFF and 100 ng/ml IL-21 (Peprotech); S3: 1 μg/ml R848, 10 ng/ml IL-2, 10 ng/ml BAFF, 100 ng/ml IL-21 and 20 ng/ml IFN-γ (Invivogen), at 37 °C, 5% CO_2_ for 11 days. Culture supernatants were tested by IgG ELISA and the cells were harvested and stained with CD19, CD27 and CD38 antibodies before acquisition on a flow cytometry.

### IgG ELISA

Culture supernatants were assessed for total IgG and anti-PvDBL-TH2 antibodies by indirect ELISA as previously reported^7^. Briefly, 1 μg/ml of anti-human IgG (clones MT91/145; Mabtech, Sweden) or 2 μg/ml PvDBL-TH2 were coated on 96-well plates followed by blocking with 5% BSA-PBS. Undiluted supernatant was added to wells followed by detection with goat anti-human IgG conjugated to horseradish peroxidase (HRP) (Sigma, USA). Signal was developed with tetramethylbenzidine (TMB) enzyme–substrate and optical density (OD) was read at 450 nm. The supernatant from non-stimulated cells were used as background of experiments.

### Statistical analysis

Statistical analyses and graphing were carried out using GraphPad Prism 8.2.1 software, GraphPad Software, USA, www.graphpad.com. Mann–Whitney *U* tests (for unpaired variables) and Wilcoxon signed rank tests (for paired variables) were performed. *P*-values < 0.05 (2-tailed) were considered statistically significant.

## Supplementary Information

Supplementary Information.
