# Supplementary material for: Interferon-γ signal drives differentiation of T-bethi atypical memory B cells into plasma cells following Plasmodium vivax infection
Source: Sci Rep. 2022 Mar 22;12:4842. doi: 10.1038/s41598-022-08976-6 (PMC8941117; doi:10.1038/s41598-022-08976-6)
Supplement: Supplementary file 1 — Supplementary Information. [file 41598_2022_8976_MOESM1_ESM.docx]

**Interferon-γ signal drives differentiation of T-bet^hi^ atypical memory B cells into plasma cells following *Plasmodium vivax* infection**

**Piyawan Kochayoo^1^, Pongsakorn Thawornpan^1^, Kittikorn Wangriatisak^1^, Siriruk Changrob^1^, Chaniya Leepiyasakulchai^1^, Ladawan Khowawisetsut^2^, John H. Adams^3^, Patchanee Chootong^1*^**

^1^Department of Clinical Microbiology and Applied Technology, Faculty of Medical Technology, Mahidol University, Bangkok, 10700, Thailand.

^2^Department of Parasitology, Faculty of Medicine Siriraj Hospital, Mahidol University, Bangkok, 10700, Thailand.

^3^Department of Global Health, University of South Florida, Tampa, Florida, 33612, USA.

*Corresponding author:

Patchanee Chootong, Ph. D.

Email. [pchooton@gmail.com](mailto:pchooton@gmail.com)

**Supplementary information**

**
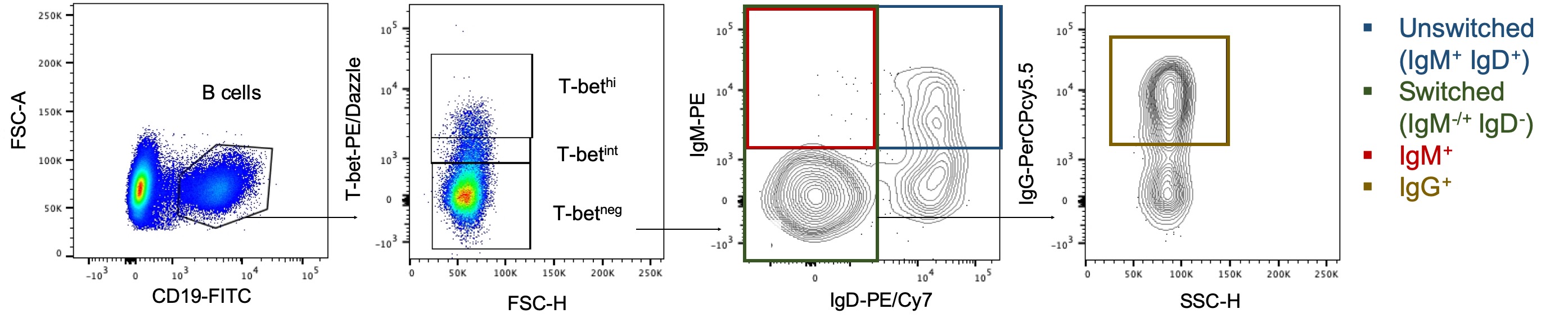
**

**Supplementary** **Fig. 1:** Representative gating strategy for class switching, and switched IgM or IgG. Among T-bet^hi^, T-bet^int^ and T-bet^neg^ B cells, the switched and unswitched MBCs were defined based on IgM and IgD expression; switched (green) (IgM^+/−^IgD^−^) and unswitched (blue) (IgM^+^, IgD^+^) MBCs. The switched IgM or IgG were defined as followed: switched IgM (red) (IgM^+^) and switched IgG (brown) (IgM^−^IgD^−^IgG^+^) MBCs.


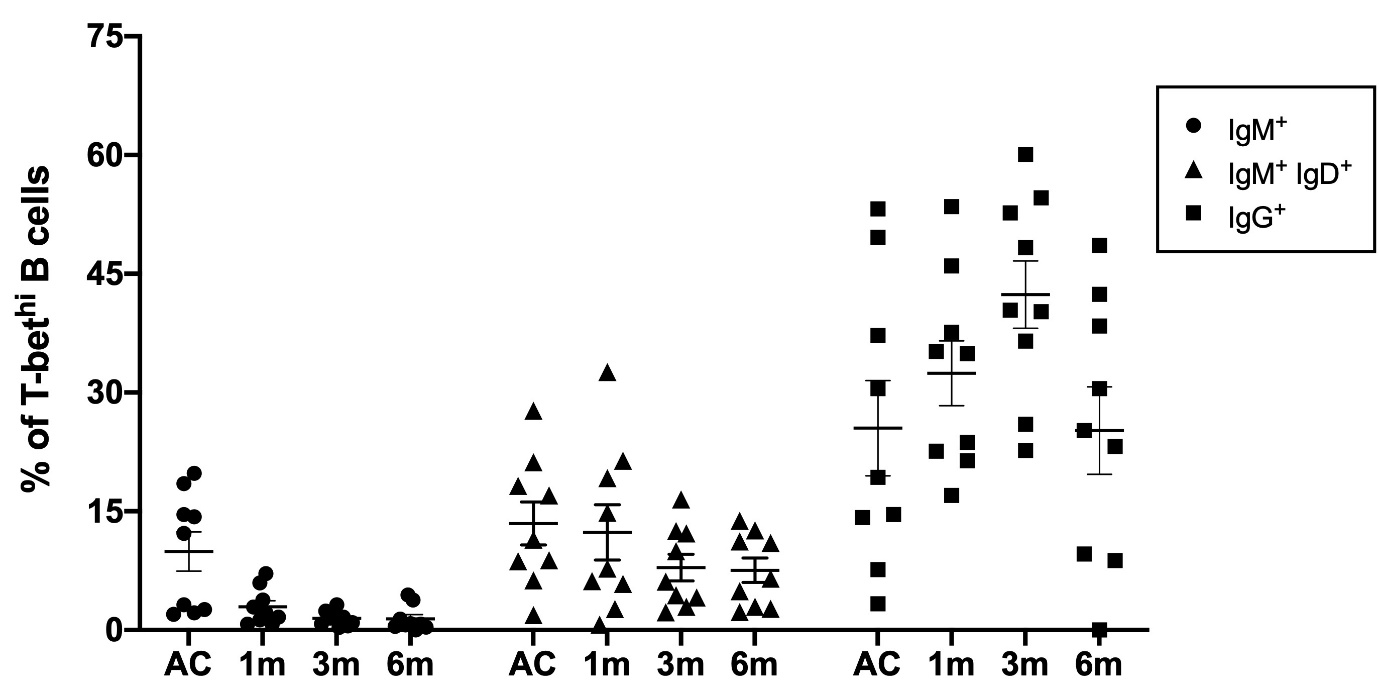


**Supplementary Fig. 2:** Kinetic responses of T-bet^hi^ unswitched (IgM^+^IgD^+^) and switched (IgM and IgG) MBCs from 9 acutely *P. vivax*-infected patients compared at three follow-up times (1, 3 and 6 months after infection). The horizontal lines represent mean values ± SEM.


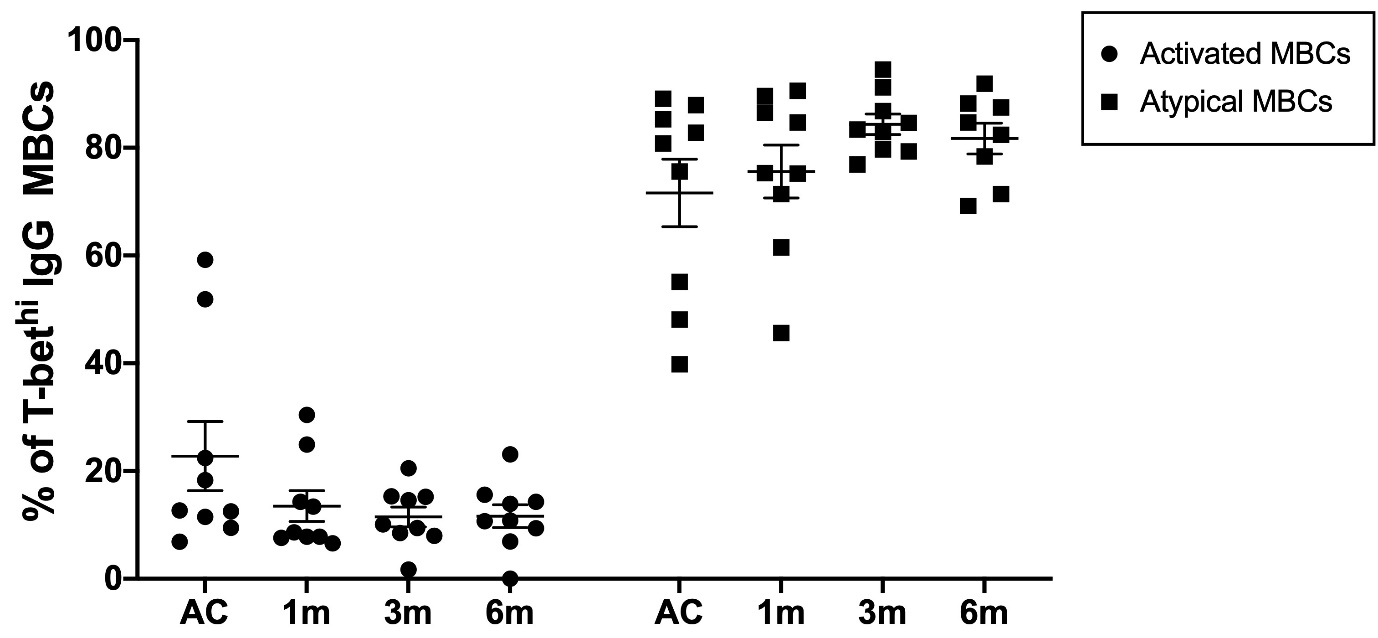


**Supplementary Fig. 3:** Kinetic responses of T-bet^hi^ IgG activated and atypical MBCs from 9 acutely *P. vivax*-infected patients compared at three follow-up times (1, 3 and 6 months after infection). The horizontal lines represent mean values ± SEM.

**
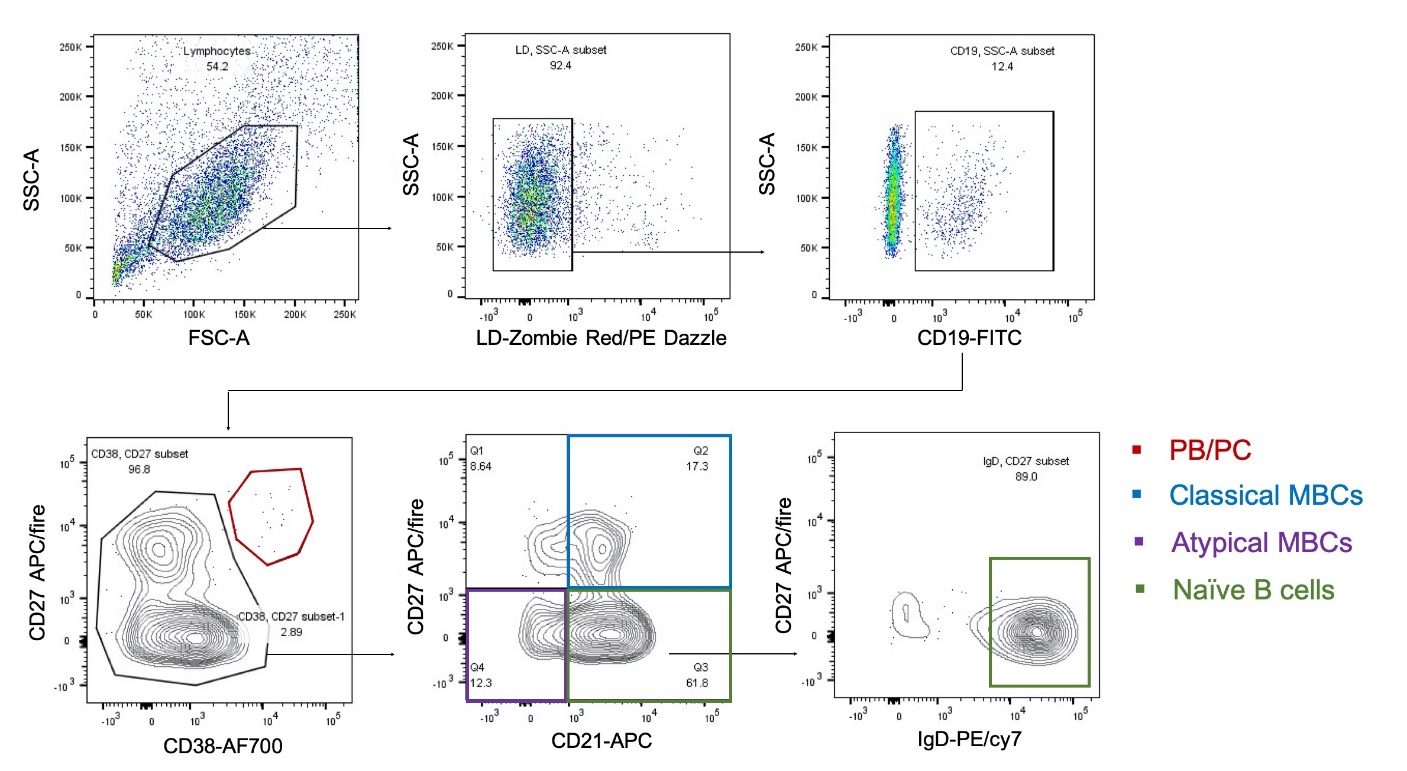
**

**Supplementary** **Fig. 4:** Representative gating strategy for MBC subset sorting. Within live CD19^+^ gate, the B cell subpopulations were defined after exclusion of plasma cells (CD27^hi^ CD38^hi^) as followed: classical MBCs (blue) (CD21^+^CD27^+^), atypical MBCs (purple) (CD21^−^ CD27^−^), naïve B cells (green) (CD21^+^CD27^−^IgD^+^). Atypical and classical MBCs were isolated to demonstrate their function in differentiation into plasma cells and secretion of antibody after *in vitro* stimulation.

| Characteristics | Acute *P. vivax* patients | | Recovery *P. vivax* subjects | | | Healthy subjects |
| --- | --- | --- | --- | --- | --- | --- |
|  | T-bet profile of  B cell subsets | T-bet^hi^ switched and unswitched MBCs | Kinetic responses | | |  |
|  |  |  | 1 month | 3 months | 6 months |  |
| Total (n) | 26 | 16 (of total 26) | 9 (of total 26) | | | 26 |
| Parasitemia (parasite/μL)  Mean ± SD (range) | 5466.81 ± 3188.75  (120 – 8,900) | | 0 | | | 0 |
| Ages  Median (IQR) | 25.50 (15.00) | 26.00 (16.00) | 18.00 (4.00) | | | 25.00 (4.00) |
| Gender |  |  |  | | |  |
| Male | 16 | 9 | 7 | 7 | 7 | 8 |
| Female | 10 | 7 | 2 | 2 | 2 | 18 |
| Nationality |  |  |  | | |  |
| Thai | 14 | 7 | 3 | 3 | 3 | 26 |
| Myanmar | 12 | 9 | 6 | 6 | 6 | 0 |
| No. of prior infection |  |  |  | | |  |
| 0 | 26 | 16 | 9 | 9 | 9 | 0 |
| 1 | 0 | 0 | 0 | 0 | 0 | 0 |
| >1 | 0 | 0 | 0 | 0 | 0 | 0 |
| No. of recorded re-infection | 0 | 0 | 0 | 0 | 0 | 0 |

**Supplementary Table S1:** Characteristics of study participants of T-bet^hi^ atypical MBC responses

|  | Marker | Fluorochrome | Clone | Catalog Number | Company |
| --- | --- | --- | --- | --- | --- |
| 1 | CD19 | FITC | HIB19 | 302206 | BioLegend |
| 2 | CD21 | APC | Bu32 | 354906 | BioLegend |
| 3 | CD27 | APC/fire | M-T271 | 356428 | BioLegend |
| 4 | CD38 | AF700 | HB-7 | 356624 | BioLegend |
| 5 | IgM | PE | MHM-88 | 314508 | BioLegend |
| 6 | IgD | PE/Cy7 | IA6-2 | 348210 | BioLegend |
| 7 | IgG | PerCP cy5.5 | M1310G05 | 410710 | BioLegend |
| 8 | T-bet | PE/Dazzle | 4B10 | 644828 | BioLegend |
| 9 | CD11c | AF700 | Bu15 | 337220 | BioLegend |
| 10 | CD69 | PE/Cy5 | FN50 | 310908 | BioLegend |
| 11 | CD86 | PE/Cy5 | IT2.2 | 305408 | BioLegend |
| 12 | CD40 | PE/Cy5 | 5C3 | 334314 | BioLegend |
| 13 | HLA-DR | APC | G-46-6 | 559866 | BD Pharmingen |
| 14 | IL-21R | PE/Cy7 | 17A12 | 359514 | BioLegend |
| 15 | FcRL4 | PE | 413D12 | 340204 | BioLegend |
| 16 | FcRL5 | PE | 509f6 | 340304 | BioLegend |
| 17 | CD95 | PE/Cy5 | DX2 | 305610 | BioLegend |
| 18 | CXCR5 | AF700 | J252D4 | 356916 | BioLegend |
| 19 | CCR7 | PE | G043H7 | 353204 | BioLegend |
| 20 | pSyK (Y352) | PE/Cy7 | 17A/P-ZAP70 | 561458 | BD Bioscience |
| 21 | pBLNK (Y84) | AF647 | J117-1278 | 558443 | BD Bioscience |
| 22 | pPLCγ2 (Y759) | PE | K-86-689.37 | 558490 | BD Bioscience |
| 23 | Zombie NIR^TM^ Fixable Viability Kit | - | - | 423105 | BioLegend |
| 24 | Zombie Red^TM^ Fixable Viability Kit | - | - | 423109 | BioLegend |

**Supplementary Table S2:** Flow cytometry panel used for this study
